# Innate Immunity to H5N1 Influenza Viruses in Humans

**DOI:** 10.3390/v4123363

**Published:** 2012-11-26

**Authors:** Irene Ramos, Ana Fernandez-Sesma

**Affiliations:** Department of Microbiology and the Emerging Pathogens Institute, Mount Sinai School of Medicine, New York, New York, USA

**Keywords:** H5N1, influenza virus, cytokines, innate immunity, virulence factors, antiviral response

## Abstract

Avian influenza virus infections in the human population are rare due to their inefficient direct human-to-human transmission. However, when humans are infected, a strong inflammatory response is usually induced, characterized by elevated levels of cytokines and chemokines in serum, believed to be important in the severe pathogenesis that develops in a high proportion of these patients. Extensive research has been performed to understand the molecular viral mechanisms involved in the H5N1 pathogenesis in humans, providing interesting insights about the virus-host interaction and the regulation of the innate immune response by these highly pathogenic viruses. In this review we summarize and discuss the most important findings in this field, focusing mainly on H5N1 virulence factors and their impact on the modulation of the innate immunity in humans.

## 1. H5N1 viruses and human infection

Influenza A viruses (IAV) are negative-sense and single-stranded RNA, enveloped viruses, belonging to the family *Ortomixoviridae*. Their genome is organized in eight RNA segments, encoding up to 13 proteins [1,2,3]. The hemagglutinin (HA) and neuraminidase (NA) are the most abundant glycoproteins in the virion. To date, 17 HA and 9 NA subtypes have been described (reviewed by [4,5]) and the combination of those proteins results in diverse subtypes of IAV.

Different subtype combinations of IAV circulated in humans during the last century. The variation in circulating subtypes is usually a consequence of reassortment (antigenic shift) that occurs in animal reservoirs, resulting in a new subtype that is able to transmit to the human population (reviewed in [6]). The circulating subtypes as of 2012 are H1N1 viruses which caused a pandemic in 2009, and H3N2 viruses. Aquatic wild birds are the natural hosts of IAV. Sixteen HA subtypes (1–16) and nine NA subtypes have been detected in different combinations in aquatic wild birds, mostly establishing short-lived subclinical enteric infections (reviewed in [7,8]). Sporadically, viruses transmit from aquatic wild birds to poultry or mammals, and new genotypes of influenza virus may become established in these new “non-natural” hosts. The most important of those influenza viruses are the H5 and H7 subtypes. Some of them evolve to become genetic variants that are able to cause severe disease in poultry and mammals. 

Since 1997 there have been several outbreaks of H5N1 influenza viruses transmitted to the human population directly from poultry, showing great virulence and low rates of survival [9,10,11,12]. These viruses are known as High Pathogenic Avian Influenza Viruses (HPAIV). Common symptoms at early stages of the infection are fever, cough and dyspnea, and in the most severe cases, development of acute respiratory distress syndrome and respiratory failure [10,12]. As of August 2012, more than 600 confirmed cases of infection by H5N1 viruses have been reported to the World Health Organization (WHO), with a lethal outcome in 59% of those documented cases [13]. However, there is evidence that indicates that this lethality rate might be overestimated due to reduced sensitivity of the WHO confirmation criteria, no formal H5N1 confirmation by health providers in rural areas, and subclinical or mild infections [14]. Fortunately, avian strains of IAV are not efficient at infecting humans [15], and direct transmission from human to human has been reported only in close family clusters, with very limited spread of the virus [16].

In this review we discuss the findings that researchers in the virology and immunology fields have reported to date regarding the induction and evasion of the innate immune response to HPAIV in humans, which is believed to contribute to the severe pathogenesis that these IAV cause in the human host. Also, we focus on the virulence factors of HPAIV that might contribute to the hyper-induction of cytokines or hypercytokinemia and/or evasion of the antiviral response. 

## 2. Hypercytokinemia Induction in Humans by H5N1: Insights from *ex vivo* Experimental Models

Humans infected by H5N1 frequently present high serum levels of pro-inflammatory cytokines and chemokines [11,17,18], which is believed to contribute to the disease pathogenesis. Acute respiratory distress syndrome (ARSD), which has been compared to the severe acute respiratory syndrome virus due to Coronavirus (SARS-CoV) and associated with exaggerated inflammatory responses (reviewed in [19]), has been frequently observed in patients infected by H5N1 IAV with severe degrees of disease [20]. However, the precise mechanisms of H5N1 infection leading to hypercytokinemia in humans are still unclear.

The first steps in the initiation of immune responses involve sensing of pathogen associated molecular patterns (PAMPs) from the invading microorganism by pattern recognition receptors (PRRs) in infected cells, including dendritic cells (DCs). This recognition initiates a series of signaling events that result in the secretion of inflammatory cytokines, type I IFN, chemokines and antimicrobial peptides, and also induces maturation of DCs (reviewed by [21]). PRRs are distributed in different sub-cellular localizations. Thus, some of them are in the plasma membrane, as is the case of the toll-like receptors (TLR) such as TLR2, TLR4, TLR5, TLR6 and some C-type lectins. Others are located and sense PAMPs in the endosomal compartments, such as TLR3, TLR7 and TLR9. Additionally, the cell presents cytoplasmic receptors, as RNA helicases, that are capable of sensing intracellular viral RNAs, like the retinoic acid inducible gene I (RIG-I) and the melanoma differentiation antigen 5 (MDA5), or the Nucleotide Oligomerization Domain (NOD)-like receptors (NLRs) (reviewed by [22,23]).

The respiratory upper and lower airways are continuously exposed to pathogens, and therefore viral interactions with PRRs in cells of these tissues are crucial to elicit innate immune responses in the host against those pathogens (reviewed in [24]). The main cell targets for influenza viruses are epithelial cells in the lungs, which express TLR3 and RIG-I [25,26], both of them important for the induction of type I IFN upon sensing of influenza virus replicative and genomic RNA [26,27,28,29,30]. Immune cells such as macrophages and DCs are also present in the respiratory system, and given their high expression of PRRs and efficiency at producing pro-inflammatory cytokines upon activation (that also induce antiviral responses in neighboring cells), they represent crucial elements of the innate immune response to pathogens. DCs, and macrophages to a lower extent, are professional antigen-presenting cells (APCs), and therefore they link innate and adaptive immune responses (reviewed in [31,32]). Among DCs, both myeloid and plasmacytoid DCs (pDCs), are resident in the human airway and lung tissues [33,34,35,36]. Myeloid DCs mainly sense influenza viruses through RIG-I and TLR3 [37,38], while plasmacytoid DCs express high levels of TLR7, which recognizes influenza virus ssRNA in the endosomes resulting in high and early induction of type I IFN [39,40,41]. The most abundant macrophage population in the lung is the alveolar macrophages, which express TLR2, TLR3, TLR4, TLR5, and TLR6 at high levels [24,42,43], as well as RIG-I, which is important for the recognition of influenza viruses [44].

The use of *ex vivo* primary human cell systems has provided interesting insights into the innate immune responses generated against IAV in general, and H5N1 in particular. Table 1 summarizes the most relevant findings reported in those types of studies. Among the most frequently used models in IAV research are the primary human tracheo-bronchial epithelial cell (HTBE) and primary bronchial epithelial cells (BECs) [45], used either non-differentiated [46] or differentiated in an air-liquid interphase. Differentiation results in pseudo-stratified and polarized cultures, containing ciliated, secretory, and basal cells that resemble human airway epithelium [47,48]. Interestingly, Matrosovich *et al*. [48], using this differentiated cell system, were able to show that avian and human influenza viruses infect different cell types as a consequence of their different receptor specificity (SAα2,3 and SAα2,6, discussed in the next section). Some research works on the evaluation of the cytokine responses in primary HTBE cells, as well as on the role of type-I and type II alveolar pneumocytes in response to human and avian IAV, have shown higher levels of cytokine production upon infection with H5N1 viruses than H1N1 in these cells [46,49]. Another study that compared the levels of cytokine production in HTBE upon infection with H5N1 or H1N1 showed different results depending on the differentiation state of the cells. In that study, non-polarized cells showed stronger IFN production in response to H5N1 viruses, while this response was reduced in the polarized cultures, probably due to lower infection and replication levels [50]. Two additional studies that compared the cytokine profile induced by H5N1 viruses and H3N2 human virus in bronchial epithelial cell lines (Calu-3) or using primary BECs, showed delayed type I IFN induction in H5N1 infected cells compared to H3N2 infected cells [45,51]. Therefore, although H5N1 viruses tend to induce higher level of cytokines in primary epithelial cells than H1N1, there is no clear consensus regarding the innate immune responses induced by IAV of different origin, which might be explained by the use of different strains and subtypes, high genotypic variability and the differential contribution of the viral proteins to the induction or evasion of the immune system. Also, in the real *in vivo* scenario, other cell types might contribute to the cytokine response induced in lung epithelial cells.

**Table 1:** Summary of cytokine induction by avian H5N1 IAV and comparison with H3N2 and H1N1 human IAV in human cell experimental models.

	Cell culture system	IAV subtype	Cytokine induction	Genes up-regulated	Refs.
**Non-immune cells**	HTBE (non polarized)	H5N1 vs H1N1	Higher in H5N1 infected cells	IFN-β, IP-10, RANTES, IL-6, MCP-1, IL-8	[46]
	Primary BECs	H5N1 vs H3N2	Attenuated in H5N1	IFN, PKR, RIG-I	[45]
	Calu-3	H5N1 vs H3N2	Attenuated in H5N1	IFN, PKR, RIG-I	[45]
	Polarized HTBE	H5N1 vs H3N2	Attenuated in H5N1	IFN-β, ISGs	[51]
	Polarized Calu-3	H5N1 vs H3N2	Attenuated in H5N1	IFN-β, ISGs	[51]
	Polarized HTBE	H5N1 vs H1N1	Attenuated in H5N1	IFN-β	[50]
	HTBE (non polarized)	H5N1 vs H1N1	Higher in H5N1 infected cells	IFN-β	[50]
	Primary Type I pneumocytes	H5N1 vs H1N1	Higher in H5N1 infected cells	IFN, IP-10, RANTES, IL-6	[49]
	Primary Type II pneumocytes	H5N1 vs H1N1	Higher in H5N1 infected cells	IFN-β, IP-10, RANTES, IL-6	[46]
	HMVEC (Primary endothelial cells)	H5N1 vs H1N1	Higher in H5N1 infected cells	IFN-β, IL-7, TNF, CCL2	[52]
	HUVEC (Primary endothelial cells)	H5N1 and H1N1	Higher in H5N1 infected cells	IFN-β, ISGs	[53]
	HTBE (non polarized)	H5N1	Induction of IP-10	IP-10	[18]
	HMVEC (Primary endothelial cells)	H5N1	Induction of IP-10	IP-10	[18]
**Immune cells**	hMDMs	H5N1 vs H1N1	Higher in H5N1 infected cells	TNF-α, MCP-1, RANTES, IP-10, IL-1β, IL-6, IFN-αβ, and TRAIL	[54]
	hMDMs	H5N1 vs H1N1	Higher in H5N1 infected cells	RIG-I, MDA5, TLR3, IFN-β, TNF-α, IP-10	[55]
	hMDMs	H5N1 and H1N1 or H3N2	Higher in H5N1 than H1N1/H3N2 infected cells	NF α, IFN-α/β,IL-1β, MCP-1, MIP-1α, RANTES, IL-12	[56]
	hMDMs	H5N1 and H1N1 or H3N2	Higher in H5N1/H3N1 than in H1N1 infected cells	TNF α, IL-6, MIP-1α, IP-10	[57]
	hMDMs	H5N1 vs H1N1	Higher in H1N1 infected cells	TNF-α, IFN-β, IFN-λ1,IFN-α, MCP-1	[58]
	hMDMs	H5N1 vs H1N1	Higher in H1N1 infected cells	TNF-α, IFN-β, IFN-λ1, IP-10	[59]
	MDDCs, mDCs and pDCs	H5N1		IFN-α, TNF-α	[60]
	pDCs	H5N1 and H1N1 or H3N2	Higher in H5N1 than H1N1/H3N2 infected cells	IFN-α, TNF-α	[61]

The innate immune responses to IAV have also been studied in endothelial cells by using primary human *ex vivo* models as well. The study by Zeng *et al*. [52] showed that primary human lung micro-vascular endothelial cells (HMVEC) support viral replication of H5N1 viruses and infection results in strong up-regulation of pro-inflammatory genes. Accordingly, other studies using Human Umbilical Vein Endothelial Cells (HUVEC) showed that H5N1 viruses induced strong inflammatory responses, mostly mediated by activation of the NF-κβ (nuclear factor kappa-light-chain-enhancer) pathway [52,53,62], supporting a possible role for endothelial cells in the hyper-induction of cytokines in infected patients.

It is well established that IAV infect human monocytes and macrophages, leading to high production of cytokines [63,64,65,66,67]. These immune cell types are critical for the induction of the innate immunity in the host. An interesting report by Lee *et al.* [55] explored the interaction between macrophages and epithelial cells after IAV infection using a cell culture system, and showed that cytokine mediators released from H5N1 infected human monocyte derived macrophages (hMDM) induced higher levels of pro-inflammatory cascades in epithelial cells than direct viral infection, supporting the important role of these phagocytic cells in the hyper-induction of cytokines in the lung observed after H5N1 IAV infection. This has been confirmed by several studies analyzing cytokine profiling of infected hMDM, that determined that H5N1 infection results in more elevated cytokine production than H1N1 viruses in these cells [54,56,57,58,59,68,69]. Additionally, a recent report by Cheung *et al*. [70] analyzed and compared the proteome variation induced by H5N1 and H1N1 viruses in hMDM, showing an early and enhanced translational activity after infection with H5N1 IAV than after H1N1 IAV infection, which might be important for the increased up-regulation of pro-inflammatory cytokine expression in H5N1 infected macrophages. On the other hand, IAV is known to induce apoptosis in human macrophages, which is also a mechanism believed to contribute to pathogenesis [57,71,72,73]. However, it is not clear if infection with H5N1 results in induction of apoptosis in a different fashion than seasonal human H1N1 or H3N2 influenza viruses, since conflicting data have been obtained in independent studies, suggesting that the ability of IAV to induce apoptosis might be strain rather than subtype dependent [57].

Human myeloid DCs are also susceptible to infection by IAV [66,74], while pDCs have been shown to be resistant [60]. Monocyte-derived DCs (MDDCs) is a well-established model to study antiviral responses in myeloid DCs, although research on the pro-inflammatory responses to H5N1 IAV strains has not been performed in sufficient depth in this system. Thitithanyanont *et al*. [60] showed that either blood isolated myeloid DCs (mDCs) or MDDCs can be infected by H5N1 IAV, resulting on the induction of high levels of IFN-α and, importantly, leading to cell death. The same study showed that pDCs, although resistant to infection, produce high levels of IFN-α in response to H5N1. Accordingly, Sandbulte *et al*. [61] compared the pDC responses to H5N1 with the ones to human IAV and observed increased IFN-α and TNF-α in H5N1 infected cells, which suggests a contribution of the resident DCs to the pro-inflammatory responses in the lungs upon H5N1 infection.

**Figure 1:**
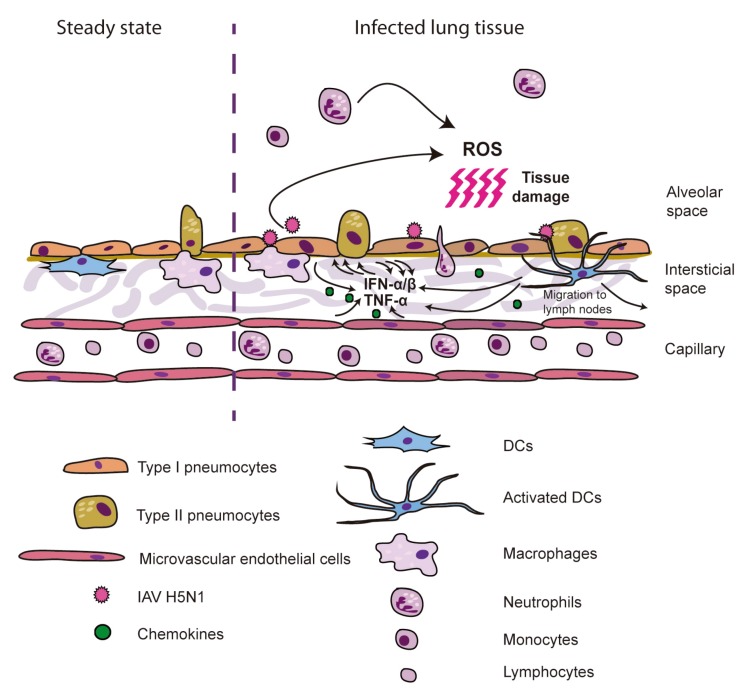
Model for ARDS induction by H5N1 IAV. H5N1 viruses infect epithelial, endothelial and inmmune cells, which upon recognition of viral PAMPs produce high levels of cytokines and chemokines. Those induce increased production of cytokines by resident cells, as well as recruitment of neutrophils, monocytes and macrophages, responsible for the oxidative stress upon production of ROS, causing severe cell and tissue damage.

Therefore, research using *ex vivo* cell models, most of them involving primary cell systems, have provided valuable information about the characteristics of the immune responses elicited by avian H5N1 viruses in human cells. These data indicate that endothelial cells and immune cells, like macrophages and DCs, produce elevated levels of cytokines and chemokines upon encounter with HPAIV. Some of those data have been recently confirmed with improved *ex vivo* tissue systems by Weinheimer *et al*. [75], such as human lung organ tissues, which showed elevated levels of cytokine production upon infection with avian IAV strains. Macrophages and DCs, due to their high expression levels of PRRs, are likely to significantly contribute to the induction of the rapid and severe damage observed in human infected by H5N1 viruses by recruiting other immune cells via chemokine production, as monocytes and neutrophils, and stimulating the cytokine amplification in epithelial and endothelial cells. These events result in the release of pro-inflammatory mediators that have seriously damaging effects in airway and lung tissues [76]. Although the mechanisms for lung injury development during acute distress syndrome are no yet well understood, it is known that the oxidative stress mediated by the release of reactive oxygen species (ROS), and other cytotoxic mediators as a consequence of an acute innate immune response play an important role [77,78,79] (Figure 1).

A question that is also under investigation by many groups in the IAV field is why H5N1 avian influenza viruses cause this cytokine burst in humans. In the subsequent sections of this review we will cover the current knowledge on the modulation of the innate immune response in the host by different proteins of HPAIV virus. Some of these proteins have been shown to be involved in eliciting immune responses, as the HA, while others, as the non-structural protein (NS) 1, are known to be very efficient at antagonizing the host immune response. 

## 3. HA: Involvement in Entry and Cell Signaling

The HA of IAV plays an essential role in initiation of infection, since it interacts with sialic acid (N-acetylneuraminic acid) receptors (SA) on the cell surface [80], which allows for subsequent endocytosis of the viral particle. It also mediates fusion of the endosomal and viral membrane (reviewed in [81]). The main host species barrier that limits transmission of avian IAV to humans consists of the different receptor specificity of avian and human viruses [82,83,84]. SA are frequently attached through α2,3 or α2,6 (SAα2,3 or SAα2,6) linkages to the terminal galactose of the underlying sugar chains of glycoproteins in the cell membrane. It is known that HA from human isolates usually binds SAα2,6, which are abundant in the human upper respiratory tract, while the one from avian isolates usually has affinity for SAα2,3 [85]. Therefore, the low abundance of SAα2,3 in the human respiratory tract is one of the limiting factors for the lack of transmission of avian influenza viruses in humans. Recently, two independent groups have been able to identify some of the amino acid changes that allow airborne transmission of avian IAV to a mammalian host using the ferret model [86,87]. As other previous reports suggested before (reviewed by [88]), the protein that bears most of the modifications necessary for transmission in mammals is the HA, as increased affinity for SAα2,6 is necessary in order to productively infect cells in the upper respiratory tract. However, these studies also confirmed that changes in the receptor specificity are not sufficient for transmission of avian IAV to humans (or mammalian systems) and *vice versa*, and that modifications in the polymerase, specifically in the PB2 component [89,90], are necessary for transmission. 

Although SAα2,6 are the most abundant in the respiratory system in humans, some cell types have been shown to present SAα2,3 glycoconjugates (therefore being susceptible to infection by H5N1 viruses) mainly in the lower respiratory tract, including type II pneumocytes and alveolar macrophages [82,83,84]. Additionally, human microvascular endothelial cells [52,91], human MDDCs [66] and human MDMs [68], present avian-like and human-like receptors on the surface, and therefore they are susceptible to infection by avian and human influenza viruses. Therefore, those cells bearing SAα2,3 receptors are relevant for initiation of infection by avian influenza viruses, and also for induction initial innate immune responses via PRRs, as RIG-I or TLR-dependent recognition. 

Interaction of the HA with cell receptors is also believed to contribute to the induction of cytokine production (reviewed in [92]). Xu *et al*. [93] showed that exposure to HA from a HPAIV H5N1 (A/chicken/Guangdong/191/04) induced the activation of the Janus Kinases (JAK) 2 and 3, as well as the transcription factors STAT (signal transducer and activator of transcription) and NF-κβ of activated B cells, which correlated with induction of IP-10, IL-6, IL-8, MCP-1, MIP-1α, MIP-1β and RANTES. Another study that supports a possible role for influenza HA in cell signaling showed that recombinant H5 and H1 induce activation of human DCs [94]). Also, a study from our group showed that the avian or human-like receptor specificity might have an effect in the activation of the pro-inflammatory responses as observed after infections of primary human MDDCs, MDMs and HTBE cells with recombinant viruses bearing HA proteins with different receptor specificities [66]. Other reports have shown induction of different signaling pathways upon interaction of IAV and receptors in the cell surface. For instance, virus-receptor interaction has been reported to activate receptor tyrosine kinases (RTKs) [95], p38 mitogen-activated protein kinase (MAPK) and extracellular signal-regulated kinase (ERK) [96].

One of the most important virulence determinants of the HPAIV is the presence of a multibasic cleavage site (MBCS) as shown in avian and mammalian models [97,98]. This polybasic amino acid sequence in the cleavage site allows post-translational processing of the precursor HA0 to HA1 and HA2, which is necessary for fusion activity and virus entry, by furin and other subtilisin-like proprotein-processing endoproteases that are ubiquitinously expressed in tissues [99,100]. Cleavage of the low pathogenic avian IAV HA, on the other hand, is limited to trypsin-like proteases with a more restricted distribution, due to the presence of a single basic amino acid in the cleavage site [101]. Although avian viruses with HA with MBCS are known to have enhanced virulence, other viral factors might be necessary for this effect in mammals, since an interesting report showed that the introduction of a MBCS sequence in a human isolate H3N2 is not sufficient to increase pathogeneicity in ferrets [102]. The main role of the MBCS in pathogenesis by IAV is by allowing increased viral infection and replication in mammals [103], but it has also been associated to increased levels of cytokine production [103], probably as a result of higher detection of viral RNA by PRRs due to the increased levels of infection.

Natural Killer (NK) cells also represent important players in the innate immunity to viral infection. Upon activation and recruitment to the site of infection, NK cells produce cytokines and destroy infected cells (reviewed by [104]). There is evidence that influenza virus HA can induce activation of NK cells, by interacting with the receptors NKp46 and NKp44 [105,106,107], which is thought to be a mechanism for killing infected cells. This interaction seems to be dependent on the sialylation of the receptor, and have been demonstrated for H5 and H1 (that bind through SAα2,3 and SAα2,6, respectively). However, NKp46 interactions with H5, as opposed to the ones with a swine origin H1 2009, are insufficient to activate the NKp46-mediated killing [108], suggesting that H5 pathogeneicity might be increased by this lack of ability to activate NK cells. On the other hand, another study showed that infection with pseudotyped particles expressing influenza virus HA induced higher levels of human NK cell activation as shown by up-regulation of the activation marker CD69 [109], and in this case H5N1 and H1N1 1918 expressing pseudo-particles induced higher levels of activation that the ones expressing an H1N1 2009 HA, which suggests different outcomes depending of the viral HA. Due to the variety of sialylated glycans that HA from different IAV strains interact with [110,111], it would be possible that those interactions have diverse outcomes depending on the IAV subtype/origin, resulting in different levels of pathogenesis. 

## 4. NS1: The Innate Immunity Suppressor

The NS1 protein from IAV is one of the strongest viral antagonists that have been described and its numerous functions have been extensively studied. However, given the complexity and great number of functional activities, inter-connection and overlapping of those functions and high strain variability, there is still much to learn about this innate immune suppressor. IAV NS1 protein has multiple different functions during cell infection, most of them related to its inhibitory effects on of type I IFN production and NF-κβ activation [112] (reviewed by [113,114]). NS1 is a dsRNA binding protein, and this feature allows it to sequester the viral RNA and prevent activation of the 2'-5' oligo (A) synthetase (OAS)/RNase L pathway and of the protein kinase RNA-activated (PKR) [115,116]. Also, the dsRNA binding domain mediates direct interaction with RIG-I, TRIM-25 and PKR, resulting on a dampened antiviral state mediated by those IFN inducible genes (ISGs) and increased viral replication [117,118,119,120]. On the other hand, the C-terminal or effector domain of NS1 presents the ability to specifically inhibit 3’ processing of cellular pre-mRNA, and therefore to reduce the protein expression of cellular proteins involved in the host cell immune response, by interacting with the 30 KDa subunit of the cleavage and polyadenylation-specificity factor (CPSF30) [121,122] and the poly(A) binding protein II [123]. Recenly, Gao *et al*. [124] described that the C-terminal domain of an NS1 from an H5N1 targets IKKα and IKKβ and therefore suppresses NF-κβ activation. Additionally, IAV NS1 has been described to hijack the host translational machinery to enhance viral translation [125,126], and can also interact with the p85β regulatory subunit of PI3K, resulting in subsequent Akt activation [127]. Another recent publication has shown that the C-terminal region of the NS1 of human H3N2 influenza viruses acts as a histone tail that mimics and inhibits host transcriptional elongation by interacting with the human PAF complex [128]. All these data together support the idea that the NS1 is a multifunctional innate immune inhibitor and is an important virulence factor for IAV.

Some functional differences have been described between NS1 from avian and human IAV, although due to the NS1 complexity it is difficult to determine if those differences might impact the innate immune response of HPAIV H5N1 in humans. The study from Kainov *et al*. [129] showed the avian IAV NS1 increased translation in an *in vitro* cell-free assay, in contrast to the NS1 from a human H1N1 2009 isolate. However, this same study showed that the H5N1 NS1 presented a stronger poly-adenylation inhibitory capacity (higher CPSF inhibition) than an NS1 from a human or low pathogenic avian virus. Human IAV present two highly conserved amino acids in the central region of the NS1 protein, F at 103 and M at 106, necessary for stabilization of the NS1-CPSF30 complex [130,131]. NS1 from avian H5N1 viruses isolated from human before 1997 do not present these amino acids and consequently they are unable to inhibit CPSF30 efficiently. However, it also has been shown that an H5N1 virus bearing an L103/I106 NS1 was also able to inhibit CPSF30 function to some extent since other viral proteins, specifically PA and NP, stabilize the NS1-CPSF30 complex [132].

CPSF30 inhibition has been associated with limitation of the production of type I IFN and pro-inflammatory cytokine production in cell culture [122,133] (unpublished observations from our laboratory). In contrast, *in vivo* studies showed that introduction of the amino acids F103/M106 in an avian H5N1 1997 isolate NS1 increases pathogeneicity, replication and cytokine production in mice [134]. So therefore, these contradictory data in cell culture vs. *in vivo* models seem to indicate that the NS1 antagonist function might be critical at early steps of the infection, allowing the virus to replicate more efficiently, which in subsequent stages of the infection *in vivo* results in increased release of type I IFN and pro-inflammatory cytokines. Interestingly, H5N1 isolated from humans after 1998 viruses present the amino acids F103/M106, correcting the defective CPSF30 binding and showing increased virulence [135].

Another feature of IAV NS1 is that the C-terminal four amino acids constitute a PDZ binding domain (PBD), as identified by Obenauer and colleagues [136] while performing a large-scale sequence analysis of avian influenza isolates. They observed that the most represented sequence in avian viruses was ESEV, while the consensus sequence for the human isolates examined was RSKV. Also, they determined that EPEV and ESEV were characteristically found in HPAIV human infections in 1997 and 2003, and KSEV in the 1918 pandemic virus. Interestingly, those motifs showed better ability to bind some known PDZ containing human proteins, and subsequent work demonstrated that these sequences are pathogenicity determinants in mice [137,138]. PDZ domains are present in a great diversity of proteins, and play important roles in multiple biological processes, such as in assembly of supra-molecular signaling complexes, protein transport and localization, and cell polarity organization (reviewed in [139]). Therefore, it is not surprising that the differential interaction of NS1 with these proteins might have multiple consequences regarding apoptosis, immune responses or viral replication. However, these processes are not well characterized yet, although some of those proteins that interact with EPEV and ESEV sequences have been already identified [140,141,142,143,144]. For instance, NS1 bearing the ESEV PBD sequence interacts with the PDZ motif of a pro-apoptotic protein named Scribble, reducing cell dead in the infected cells [144]. Also, it has been recently shown that the avian influenza NS1 protein bind and stimulate human Src tyrosine kinase through their C-terminal Src homology type 3-binding (SHB) domain. Further and ongoing research will provide better insights regarding the outcome of the HPAIV NS1 PBDs interaction with PDZ-containing proteins. 

**Figure 2:**
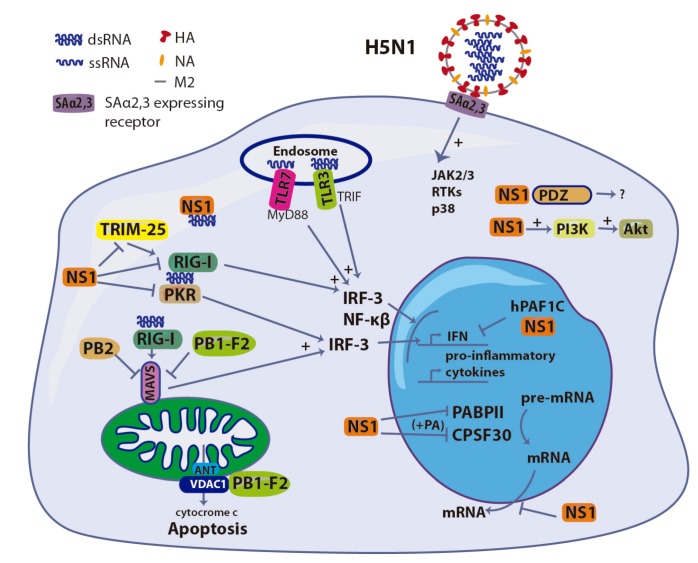
Induction and antagonist role of different components of H5N1 IAV in the innate immune response in the host-cell. ssRNA, dsRNA and HA participate in the induction of the production of IFN and pro-inflammatory cytokines, while NS1, PB1-F2, PB2 and PA have an antagonist effect.

In summary, NS1 IAV is an innate immunity antagonist, which modulates the immune response in different and complex ways. The role of this protein in enhanced pro-inflammatory responses in HPAIV infected humans might be related with an early inhibition of the antiviral response that, together the interaction with PDZ-containing proteins, would lead to enhanced viral replication. *In vivo*, this enhanced replication would lead to an abrupt pro-inflammatory response upon recognition of viral RNA by PRRs in epithelial and endothelial cells and resident DCs and macrophages. A summary of these NS1 functions at the cellular level, as well as the role of other IAV components discussed in other sections of this review, are depicted in Figure 2.

## 5. PB1-F2: A Double-edged Sword

PB1-F2 is a small protein encoded in the +1 reading frame of PB1 [145] that localizes to the mitochondria and has been shown to induce apoptosis in infected cells, specifically in immune cells, by altering the mitochondrial membrane potential [145,146,147]. PB1-F2 expression increases virulence in mice [148], and it has been also reported to increase susceptibility to secondary bacterial infections in animal models [149]. Along the same lines, a serine in position 66 (instead of an arginine), has been associated with increased pathogeneicity, as shown by Conenello *et al.* [150] using recombinant viruses bearing a 1918 H1N1 or an H5N1 IAV PB1 segment. It is important to note that the PB1-F2 protein is not expressed by all the IAV. In many swine and human isolates, protein translation is interrupted because stop codons are introduced in the reading frame [145,151]. On the contrary, PB1-F2 is highly conserved in H5N1 avian influenza viruses [151] and the residue 66S is present in some of the HPAIV [150]. Therefore, the expression of PB1-F2 and the presence of the amino acid 66S are considered virulence markers, which is critical for surveillance purposes. Interestingly, the pandemic H1N1 that jumped into the human population in 2009 did not expressed PB1-F2, correlating with a low virulent profile. This is an excellent example of how the research on influenza pathogenesis can provide important and useful information regarding the threats that we might face in a pandemic situation. Related to this, an important fact to take into account when considering PB1-F2 as a virulence factor, is that its pathogenic potential is strain-specific. Thus, in the case of the H1N1 2009 pandemic virus, Hai *et al*. [152] showed that the modification of the H1N1 2009 pandemic virus in order to allow the expression of PB1-F2 had a very slight impact in pathogenesis in mice. 

Zamarin *et al*. [147] unraveled the mechanism by which this small protein induces apoptosis. They showed that PB1-F2 interacts with the adenine nucleotide translocator 3 (ANT3) and the membrane voltage-dependent anion channel 1 (VDAC1) of the mitochondrial permeability transition pore complex (PTPC). They also found that PB1-F2 also renders the cells more sensitive to pro-apoptotic stimuli, like TNF-α. On the other hand, other group showed that PB1-F2 can also oligomerize to form pores in the mitochondrial membrane [153].

In addition, another virulence mechanism for PB1-F2 has been recently identified and characterized, namely the ability of PB1-F2 to inhibit type I IFN production both in cell culture and *in vivo* [150,154]. The C-terminal domain of PB1-F2 interacts with the mitochondrial antiviral signaling (MAVS) protein, decreasing the mitochondrial membrane potential and resulting in the inhibition of RIG-I-mediated of IFN-α/β production [155]. Data from our laboratory contributed to a report that confirmed the effect of the 66S residue on the inhibition of type I IFN in human primary DCs, indicating a potential important contribution to the pathogenesis of PB1-F2 66S in the human host [154]. Due to the small size of PB1-F2 and wondering how such a small protein may have such a strong impact on two important cell events such as apoptosis induction and IFN production, it has been suggested that those two functions might be linked at the level the MAVS protein [156].

Therefore, the PB1-F2 protein, expressed by most of the HPAIV H5N1, is believed to have an important impact on the innate immune response in humans by two different processes. One the one hand, it promotes apoptosis of immune cells and, on the other hand, it inhibits IFN production at early stages after infection. So, as it may be the case of the NS1 protein, this might result in early inhibition of the antiviral state followed by and increased replication and subsequent hypercytokinemia in infected humans.

## 6. Polymerase

Another element from IAV known to be involved in the deregulation of the antiviral immune response is the polymerase complex. One of the polymerase components, the PB2 bearing a lysine in position 627, which is predominant in human IAV and in some HPAIV strains, is considered a virulence determinant in mammals for H5N1 IAV, since viruses bearing this residue are more virulent and pathogenic in mice than those with a glutamic acid, encoded in PB2 from low pathogenic avian IAV strains [157]. Interestingly, 627K has been also demonstrated to be associated with strong production of TNF-α, IFN-β and IP-10 in human primary macrophages and type 1–like pneumocytes infected with HPAIV H5N1, and therefore is thought to contribute to the elevation of cytokines in serum in H5N1 infected patients [158], although the mechanisms for this phenotype are yet unknown.

Nevertheless, components of the polymerase complex are also involved in the evasion of the innate immunity. Each component of the viral polymerase complex, PA, PB1 and PB2, has been shown to interact with MAVS. However, PB2 has the strongest MAVS-induced IFN-α/β production inhibitory ability [159]. This function seems to be common to H1N1 and H5N1 viruses isolated from humans, but H5N1 isolated from birds does not have mitochondrial localization, and therefore do not associate with MAVS, due to a polymorphism in the amino acid position 9, indicating that the PB2 mitochondrial localization might be necessary for human host adaptation of H5N1 [160].

IAV polymerase also contributes to the host protein shut-off by two independent mechanisms. On the one hand, it indirectly participates in the evasion of the antiviral response by supporting the NS1 protein in the inhibition of CPSF30, as discussed above (Section 4). Kuo and Krug [132] showed that PA is able to stabilize the NS1-CPSF30 complex of an H5N1 NS1 lacking the residues F103/M106, which allows the inhibition of the post-transcriptional processing of cellular pre-mRNA. On the other hand, it is known that, in order to hijack the host transcriptional system, IAV polymerase associates with the host RNA polymerase II (Pol II). It has been described that this interaction results in the degradation and inhibition of Pol II, which seems to contribute to the inhibition of the cellular gene expression and therefore, of the antiviral response [161] (reviewed in [162]).

Hence, the influenza polymerase proteins have been associated both with hyper-induction of the inflammatory response and with evasion of the innate immunity, and therefore contribute to the idea that the “cytokine storm” induced in humans upon infection with HPAIV H5N1 might be a consequence of a compendium of altered innate immune functions at different times and at different levels.

## 7. Concluding Remarks

**Figure 3:**
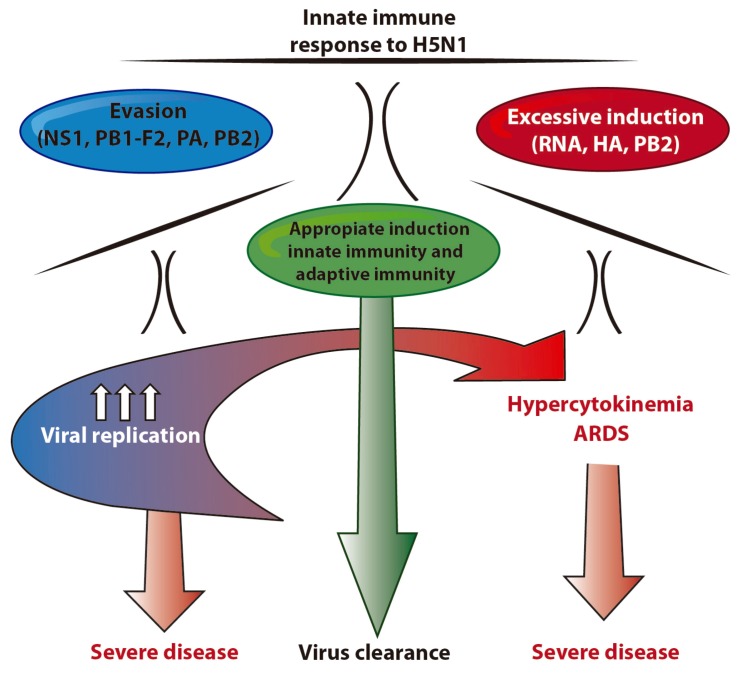
Induction of hypercytokinemia by H5N1 influenza viruses results from an imbalanced innate immune response. H5N1 influenza viruses are able to dysregulate the innate immune response, either by inducing high production of cytokines or by inhibiting the antiviral response. The evasion of the innate immunity might have as a consequence increased levels of viral replication, which contributes to increased PAMPs detection and cytokine induction. A combination of these events *in vivo* might explain how H5N1 innate immunity antagonists contribute to hypercytokinemia development in humans.

HPAIV H5N1 viruses induce severe disease in humans, probably as a consequence of the induction of an exacerbated innate immune response. Research using *ex vivo* cell models indicates that H5N1 IAV induce a high concentration of cytokines and chemokines in primary endothelial cells, as well as in immune cell types such as macrophages and DCs, indicating that the strong cytokine induction might be mediated by those cells. H5N1 components have been involved in both induction and inhibition of the innate immune response, and for that reason the contribution of these different elements at different stages of the infection *in vivo* could lead to failure of the innate immune response to control virus spread and a subsequent tissue damaging pro-inflammatory burst. In Figure 3 we show that while the desired outcome of infection is viral clearance, in the case of H5N1 IAV infections the balance is tilted due to higher virus replication and cytokine production, which result in more severe disease.

The acute pathology and severe disease related to H5N1 infection are thought to be a consequence of the hypercytokinemia observed in patients, suggesting that treatment with immuno-modulator drugs might help to diminish tissue damage and mitigate symptoms. The use of steroids has been proposed, however there is no empirical evidence that indicates benefits of this type of anti-inflammatory therapy, and dosage levels should be administered carefully, since high doses might be detrimental instead of beneficial (reviewed in [163,164]). The treatment of choice for influenza H5N1 viruses up to date is oseltamivir, which has been shown to be effective when administered early after infection, particularly before respiratory failure [164]. Nevertheless, there is a great need for new and optimized antiviral treatments or drug combinations that work more efficiently, especially in those patients diagnosed in late stages of the disease.
